# Racial and Ethnic Disparities in COVID-19 Treatments in the United States

**DOI:** 10.1007/s40615-024-01942-0

**Published:** 2024-02-26

**Authors:** Essy Mozaffari, Aastha Chandak, Alpesh N. Amin, Robert L. Gottlieb, Andre C. Kalil, Vishnudas Sarda, Mark Berry, Gina Brown, Jason F. Okulicz, Chidinma Chima-Melton

**Affiliations:** 1 Gilead Sciences, Foster City, CA USA; 2 Certara, New York, NY USA; 3 University of California, Irvine, CA USA; 4 Baylor University Medical Center, Dallas, TX USA; 5 Baylor Scott & White Heart and Vascular Hospital, Dallas, TX USA; 6 Baylor Scott & White The Heart Hospital, Plano, TX USA; 7 Baylor Scott & White Research Institute, Dallas, TX USA; 8 University of Nebraska Medical Center, Omaha, NE USA; 9 Certara, Secunderabad, India; 10 Department of Medicine, Division of Pulmonary & Critical Care Medicine, UCLA Health System, David Geffen School of Medicine at University of California, Los Angeles, CA USA

**Keywords:** COVID-19, Race/ethnicity, Treatments, Health disparities

## Abstract

**Introduction:**

Racial and ethnic disparities in patient outcomes following COVID-19 exist, in part, due to factors involving healthcare delivery. The aim of the study was to characterize disparities in the administration of evidence-based COVID-19 treatments among patients hospitalized for COVID-19.

**Methods:**

Using a large, US hospital database, initiation of COVID-19 treatments was compared among patients hospitalized for COVID-19 between May 2020 and April 2022 according to patient race and ethnicity. Multivariate logistic regression models were used to examine the effect of race and ethnicity on the likelihood of receiving COVID-19 treatments, stratified by baseline supplemental oxygen requirement.

**Results:**

The identified population comprised 317,918 White, 76,715 Black, 9297 Asian, and 50,821 patients of other or unknown race. There were 329,940 non-Hispanic, 74,199 Hispanic, and 50,622 patients of unknown ethnicity. White patients were more likely to receive COVID-19 treatments, and specifically corticosteroids, compared to Black, Asian, and other patients (COVID-19 treatment: 87% vs. 81% vs. 85% vs. 84%, corticosteroids: 85% vs. 79% vs. 82% vs. 82%). After covariate adjustment, White patients were significantly more likely to receive COVID-19 treatments than Black patients across all levels of supplemental oxygen requirement. No clear trend in COVID-19 treatments according to ethnicity (Hispanic vs. non-Hispanic) was observed.

**Conclusion:**

There were important racial disparities in inpatient COVID-19 treatment initiation, including the undertreatment of Black patients and overtreatment of White patients. Our new findings reveal the actual magnitude of this issue in routine clinical practice to clinicians, policymakers, and guideline developers. This is crucial to ensuring equitable and appropriate access to evidence-based therapies.

**Supplementary Information:**

The online version contains supplementary material available at 10.1007/s40615-024-01942-0.

## Introduction

Long-standing differential access to goods, services, and opportunities according to race and ethnicity has led to widespread and well-documented disparities in health status and medical care in the United States (US) [1, 2]. Historically and currently stigmatized groups face statistically worse health outcomes than that of White patients due to the downstream effects of structural racism, such as the widening economic divide, poor access to quality healthcare and education, systemic issues in policing and incarceration practices, poor air quality, occupational risks, and housing inequity [3].

The COVID-19 pandemic has magnified these racial and ethnic disparities, with heightened risk for COVID-19 incidence, morbidity, and mortality demonstrated in many minority populations [2].

While the Coronavirus Aid, Relief, and Economic Security (CARES) Act (2020) attempted to minimize inequity by providing relief payments to individuals, small businesses, and health care providers, the result was incomplete and inconsistent [4]. For example, hospitals serving the most privileged communities received more funds than those hospitals serving predominantly low-income minority populations [4].

The rate of hospitalization of Black patients due to COVID-19 has been shown to be more than three times higher, and for Hispanic patients over four times higher, compared to White patients [2]. Although genome-wide association studies and other genetic analyses have identified loci that are associated with adverse COVID-19 outcomes, there is no broad biological or genetic basis for the observed racial or ethnic disparities [5]. Instead, these disparities likely have their origins in social, non-biologic constructs related to systemic structural racism which has led to greater comorbidity burden in these populations and reduced access to healthcare, including access to COVID-19 vaccinations [6]. An example of the Inverse Hazard Law, the inverse correlation between power, resources, and workplace hazards was seen during the pandemic[7]. Occupations centered on in-person attendance crucial for daily business operations were predominantly occupied by Black and Hispanic workers, whereas white workers were more frequently found in white-collar professions that often came with perks like remote work reducing exposure to virus transmission [7].

Healthcare providers implicit racial biases as a result of systemic racism and the resulting normalization of discriminatory beliefs against racial and ethnic minority groups has been demonstrated to manifest in clinical decision-making. For example, racial and ethnic inequalities persist even in outpatient COVID-19 treatment. During April–July 2022, the percentage of COVID-19 adult patients treated with nirmatrelvir was 36% and 30% lower among Black and Hispanic patients than among White and non-Hispanic patients, respectively. These disparities existed among all age groups and patients with immunocompromising conditions [8].

For appropriate severity of COVID-19, dexamethasone, remdesivir, and baricitinib have important beneficial effects among selected hospitalized COVID-19 patients and are widely recommended in clinical guidelines [9–12]; tocilizumab results have been equivocal but are included in guidelines. Differences in access and delivery of these COVID-19 therapies are likely to contribute to an unnecessary and preventable exacerbation in racial and ethnic health disparities. Overtreatment in contrast to guidelines, such as use of corticosteroids or other immunomodulatory agents among patients not on supplemental oxygen, may additionally have adverse consequences.

There is a necessity to examine and identify differences in the delivery of appropriate and potentially life-saving COVID-19 therapies according to race and ethnicity, so efforts can be made to ensure the delivery of equitable healthcare and lessen inequities in COVID-19 outcomes.

The aim of the study was to characterize racial and ethnic disparities in the administration of evidence-based COVID-19 treatments among patients hospitalized for COVID-19.

## Methods

### Study Design and Setting

This is a retrospective cohort study comparing COVID-19 treatment initiation among patients hospitalized for COVID-19 in the US between 1st May 2020 and 30th April 2022 according to race and ethnicity. This study period encompasses both the pre- and post-vaccination period of the pandemic as well as the time horizon when the most severe COVID-19 mitigating interventions (e.g., lockdowns and travel restrictions) were implemented and subsequently removed. Patient-level hospitalization records were extracted from the US PINC AI Healthcare Database, a comprehensive all-payer hospital administrative dataset that captures information on inpatient discharges. The database captures data for approximately 25% of all hospitalizations occurring in 954 hospitals in 48 states in the US, accounting for over 135 million visits since it was established in 2012.

The study population is comprised of patients aged 18 years and older who were hospitalized for COVID-19, present on admission, defined as an admission with an International Classification of Diseases, 10th revision, Clinical Modification diagnosis code of U07.1. Patients were excluded if they met any of the following criteria (1) pregnant, (2) incomplete or erroneous data, (3) transferred from hospice or another hospital (4) unknown gender (5) admitted with no supplemental oxygen (NSOc) on admission to a hospital that did not report any low flow oxygen (LFO) and (6) patients requiring extracorporeal membrane oxygenation (ECMO) in the first two days of hospitalization. Exclusion criteria #5 was required since some hospitals do not bill separately for supplemental oxygen administration and instead include these costs in room charges. Since it would not have been possible to identify supplemental oxygen use in these hospitals and so only patients admitted to hospitals that reported separate charges for supplemental oxygen were included in the study. The NSOc group has been previously validated as indicative of the group of patients at lowest risk of inpatient mortality [13].

Patients were categorized according to their recorded race (White, Black, Asian, Other) and their recorded ethnicity (Hispanic, non-Hispanic, Unknown). The other race category includes race designations that were assigned by the data provider to ensure that the dataset conforms with the Health Insurance Portability and Accountability Act (HIPAA) as well as for race designations of “unable to determine.”

The outcome measured was initiation of any guideline-recommended COVID-19 treatments within two days of hospitalization, defined as documentation of corticosteroid, remdesivir, baricitinib, and tocilizumab administration [14, 15]. These are treatments recommended for use among hospitalized COVID-19 patients stratified according to their supplemental oxygen requirement. Though recommendations have evolved over time, the current National Institutes of Health (NIH) clinical guidelines recommend corticosteroids for use in patients requiring supplemental oxygen but not for NSOc patients [14]. Remdesivir is recommended for initiation among NSOc patients at high risk of progressing to severe COVID-19, among patients requiring low flow oxygen (LFO), and as an adjunct to immunomodulatory agents including corticosteroids for certain patients requiring high flow oxygen (HFO)/non-invasive ventilation (NIV). Oral baricitinib and intravenous tocilizumab are recommended for use among patients requiring LFO and who have rapidly increasing oxygen needs and systemic inflammation as well as patients requiring HFO, NIV, invasive mechanical ventilation (IMV), or ECMO. Initiation of each of these guideline-recommended COVID-19 treatments was also examined separately. Baseline was defined as the first two days of hospitalization. Covariates extracted included patient demographics (age, gender, primary payer), Charlson Comorbidity index (CCI), renal disease, immunocompromised conditions, hospital characteristics (bed size, urban/rural, teaching, region of the hospital), admission month, intensive care unit (ICU)/general ward admission at baseline, and baseline disease severity level identified through required level of supplemental oxygenation use at baseline. Baseline supplemental oxygenation use was categorized as: NSOc, LFO, HFO/NIV, and IMV. Full definitions of the baseline covariates are provided in Supplementary Table 1.

### Statistical Analysis

Logistic regression models were used to derive adjusted odds ratios (aORs) and 95% confidence intervals (CI) for the likelihood of receiving any COVID-19 treatment within 2 days of hospitalization according to race and ethnicity, separately. Models were adjusted for age, gender, hospital size, rural/urban, teaching hospital status, geographic region, primary payer, admission month/variant time period, renal disease, immunocompromised conditions, categories of CCI, and hospital ward upon admission. The models included a random intercept for hospital-level effects. All analyses were stratified according to baseline supplemental oxygen use.

## Results

**Fig. 1:**
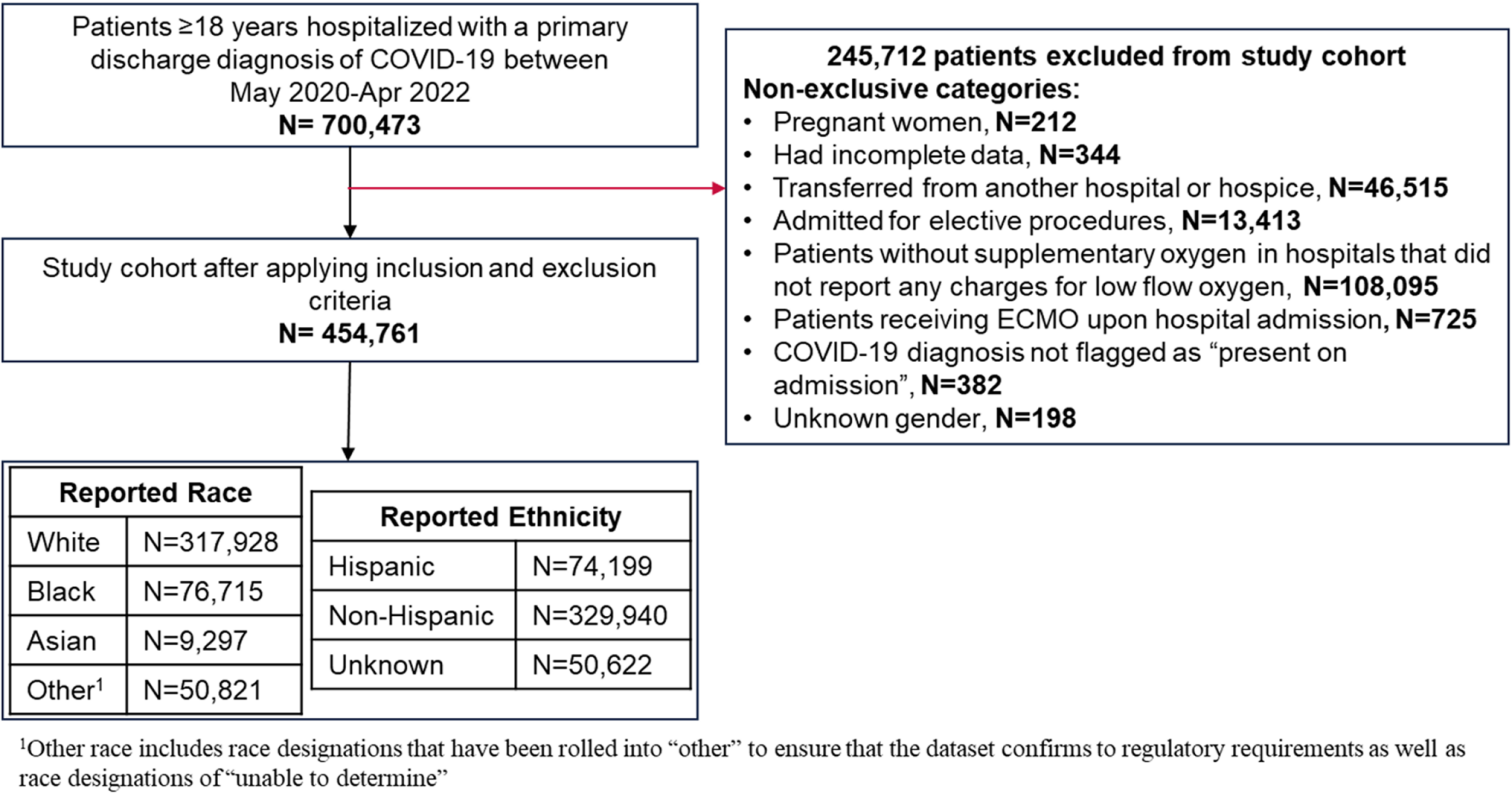
Study population

There were 454,761 eligible adults hospitalized for COVID-19 between May 2020 and April 2022 (White: 317,928 (70%), Asian: 9297 (2%), Black: 76,715 (17%), Other: 50,821 (11%)) (Fig. 1, Study Flow). The majority of the study population were non-Hispanic (N = 329,940, 72.6%), 74,199 (16.3%) patients were Hispanic and 50,622 (11.1%) were of unknown ethnicity. Table 1 presents patient baseline characteristics overall and by race. White patients were older, less likely to be admitted during the pre-Delta period, and had a lower comorbidity burden than Black, Asian, and other patients. A higher proportion of White patients received any COVID-19 treatment within 2 days of hospitalization compared to Black, Asian, and Other patients (87% vs. 81% vs. 85% vs. 84%). White patients were more likely to receive corticosteroids compared with Black, Asian, and Other patients (85% vs. 79% vs. 82% vs. 82%). White patients were less likely than Asian patients to have remdesivir (53% vs. 56%) initiated but considerably more likely than Black patients (53% vs. 43%).

**Table 1:** Baseline characteristics of study cohort overall and according to race

		Overall	White	Black	Asian	Other^1^
# Patients		**454,761**	**317,928**	**76,715**	**9297**	**50,821**
Age (years)	Median (IQR)	64 (52–75)	66 (54–77)	60 (48–70)	63 (50–74)	58 (46–70)
Age group	18–49 years	95,726 (21%)	55,945 (18%)	21,212 (28%)	2286 (25%)	16,283 (32%)
	50–64 years	137,891 (30%)	91,784 (29%)	26,635 (35%)	2781 (30%)	16,691 (33%)
	65 + years	221,144 (49%)	170,199 (54%)	28,868 (38%)	4230 (46%)	17,847 (35%)
Gender	Male	235,938 (52%)	168,528 (53%)	34,150 (45%)	5000 (54%)	28,260 (56%)
Ethnicity	Hispanic	74,199 (16%)	48,392 (15%)	1209 (2%)	193 (2%)	24,405 (48%)
	Non-Hispanic	329,940 (73%)	235,449 (74%)	69,085 (90%)	8017 (86%)	17,389 (34%)
	Unknown	50,622 (11%)	34,087 (11%)	6421 (8%)	1087 (12%)	9027 (18%)
Primary payer	Commercial	130,807 (29%)	90,562 (29%)	21,713 (28%)	3196 (34%)	15,336 (30%)
	Medicare	229,982 (51%)	173,505 (55%)	35,174 (46%)	3749 (40%)	17,554 (35%)
	Medicaid	49,919 (11%)	25,944 (8%)	12,733 (17%)	1640 (18%)	9602 (19%)
	Other payer	44,053 (10%)	27,917 (9%)	7095 (9%)	712 (8%)	8329 (16%)
Hospital location	Urban	390,518 (86%)	267,645 (84%)	68,695 (90%)	8578 (92.3%)	45,600 (90%)
	Rural	64,243 (14%)	50,283 (16%)	8020 (11%)	719 (7.7%)	5221 (10%)
Hospital bed size	< 100	32,519 (7%)	26,218 (8%)	3024 (4%)	459 (5%)	2818 (6%)
	100–199	77,214 (17%)	57,201 (18%)	10,065 (13%)	1498 (16%)	8450 (17%)
	200–299	91,302 (20%)	64,310 (20%)	14,761 (19%)	2055 (22%)	10,176 (20%)
	300–399	87,621 (19%)	57,879 (18%)	17,448 (23%)	1776 (19%)	10,518 (21%)
	400–499	43,399 (10%)	32,389 (10%)	4954 (7%)	781 (8%)	5275 (10%)
	> = 500	122,706 (27%)	79,931 (25%)	26,463 (35%)	2728 (29%)	13,584 (27%)
Hospital teaching status	Yes	178,769 (39%)	119,272 (38%)	34,259 (45%)	4406 (47%)	20,832 (41%)
Hospital region	Midwest	96,899 (21%)	73,910 (23%)	14,246 (19%)	1568 (17%)	7175 (14%)
	Northeast	43,196 (10%)	26,858 (8%)	6644 (9%)	1335 (14%)	8359 (16%)
	South	251,394 (55%)	173,269 (55%)	51,872 (68%)	2651 (29%)	23,602 (46%)
	West	63,272 (14%)	43,891 (14%)	3953 (5%)	3743 (40%)	11,685 (23%)
Admission month	Pre-Delta	273,143 (60%)	185,344 (58%)	48,137 (63%)	6470 (70%)	33,192 (65%)
	Delta	150,997 (33%)	109,850 (37%)	23,905 (31%)	2380 (26%)	14,862 (29%)
	Omicron	30,621 (7%)	22,734 (7%)	4673 (6%)	447 (5%)	2767 (5%)
CCI	0	138,559 (31%)	96,998 (31%)	19,829 (26%)	3264 (35%)	18,468 (36%)
	1 to 3	228,941 (50%)	161,816 (51%)	37,686 (50%)	4517 (49%)	24,922 (49%)
	≥ 4	87,261 (19%)	59,114 (19%)	19,200 (25%)	1516 (16%)	7431 (15%)
Key comorbidities	Immunocompromised condition	47,643 (11%)	34,604 (11%)	8205 (11%)	743 (8%)	4091 (8%)
	Renal disease	87,370 (19%)	58,103 (18%)	20,343 (27%)	1637 (18%)	7287 (14%)
Baseline supplemental oxygen requirements	NSOc	201,934 (44%)	135,059 (43%)	40,276 (53%)	4402 (47%)	22,197 (44%)
	LFO	173,143 (38%)	126,067 (40%)	23,789 (31%)	3366 (36%)	19,921 (40%)
	HFO/NIV	67,086 (15%)	48,543 (15%)	10,398 (14%)	1224 (13%)	6921 (14%)
	IMV	12,598 (3%)	8259 (3%)	2252 (3%)	305 (3%)	1782 (4%)
Baseline ICU admission		82,793 (18%)	57,604 (18%)	13,916 (18%)	1473 (16%)	9800 (20%)
COVID-19 treatment initiation upon hospital admission	Any COVID-19 treatment	390,344 (86%)	277,622 (87%)	61,927 (81%)	7895 (85%)	42,900 (84%)
	Corticosteroids	380,622 (84%)	271,155 (85%)	60,304 (79%)	7633 (82%)	41,530 (82%)
	Remdesivir	234,386 (56%)	169,695 (53%)	33,344 (44%)	5166 (56%)	26,181 (52%)
	Baricitinib	16,378 (4%)	12,672 (4%)	2001 (3%)	195 (2%)	1510 (3%)
	Tocilizumab	17,928 (4%)	12,549 (4%)	2955 (4%)	362 (4%)	2062 (4%)

^1^Other race includes race designations that have been rolled into “other” to ensure that the dataset confirms to regulatory requirements as well as race designations of “unable to determine.” See Supplementary Table 1 for variable definitions (i.e., renal disease, immunocompromised condition)

*CCI*, Charlson Comorbidity Index; *IQR*, inter-quartile range; *NSOc*, supplemental oxygen charges; *LFO*, low-flow oxygen; *HFO/NIV*, high-flow oxygen/non-invasive ventilation; *IMV*, invasive mechanical ventilation

Compared with all other races, White patients on NSOc were more likely to receive any COVID-19 treatment (White: 77.9%, Asian: 74.9%, Black: 71.2%, Other: 73.4%), corticosteroids (White: 75.4%, Asian: 71.1%, Black: 71.2%, Other: 73.4%), and baricitinib (White: 1.4%, Asian: 0.4%, Black: 0.9%, Other: 1.0) (Supplementary Table 2). Among patients requiring LFO, a smaller proportion of Black patients had remdesivir initiated than White or Asian patients (Black: 50.7%, White: 58.6%, Asian: 62.4%). This finding was also observed among patients requiring HFO/NIV (Black: 60.0%, White: 66.4%, Asian: 74.6%) and patients requiring IMV (Black: 45.3%, White: 51.7%, Asian: 56.7%).

**Fig. 2:**
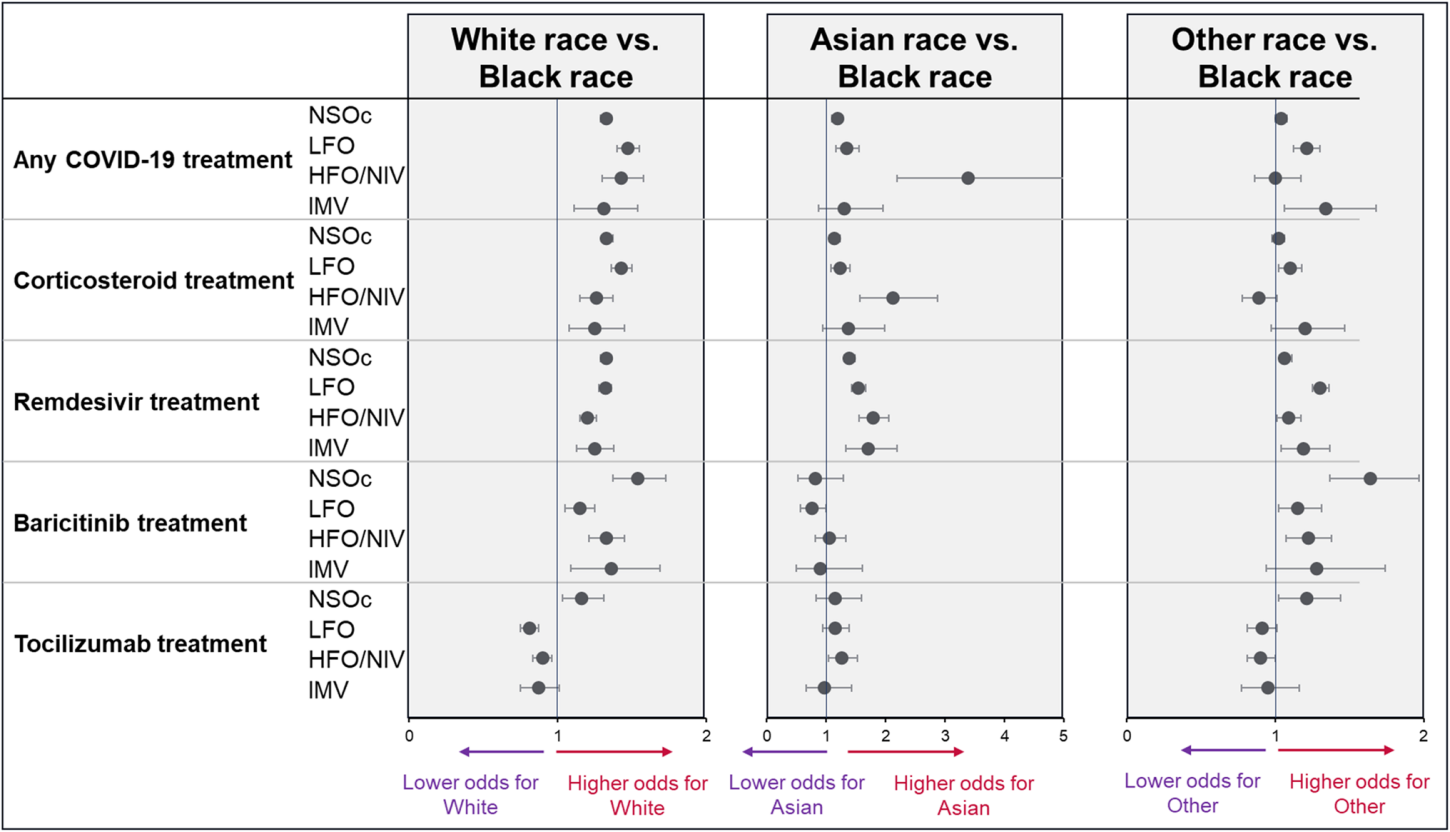
Likelihood of receiving COVID-19 treatment upon hospital admission by race (adjusted multivariable regression model). Model adjusted for age group, gender, ethnicity, hospital bed size, hospital location, teaching hospital, hospital region, payer type, variant period, CCI categories, ICU use at baseline, renal disease at baseline, immunocompromised condition at baseline. *NSOc: no supplemental oxygen charges; LFO: low-flow oxygen; HFO/NIV: high-flow oxygen/non-invasive ventilation; IMV: invasive mechanical ventilation*

In multivariable analyses, White patients were significantly more likely to receive any COVID-19 treatment than Black patients across all supplemental oxygen levels (NSOc: aOR: 1.33 (95% CI: 1.29–1.36), LFO aOR: 1.47 (1.40–1.55), HFO/NIV aOR: 1.43 (1.30–1.58), IMV aOR: 1.31 (1.11–1.54)) (Fig. 2, Supplementary Table 3). White patients were also statistically significantly more likely than Black patients to receive corticosteroids, remdesivir, and baricitinib treatment across all baseline supplemental oxygen levels, including among patients on NSOc. In contrast, White patients requiring LFO, HFO/NIV, or IMV were less likely to receive tocilizumab treatment than Black patients (LFO aOR: 0.81 (0.75–0.87), HFO/NIV aOR: 0.90 (0.83–0.96), IMV aOR: 0.87 (0.75–1.01)). Asian patients were more likely to receive remdesivir treatment than Black patients across all supplemental oxygen requirements (NSOc aOR: 1.39 (1.30–1.48), LFO aOR: 1.54 (1.43–1.67), HFO/NIV aOR: 1.79 (1.56–2.06), IMV aOR: 1.71 (1.33–2.20)). Asian patients requiring LFO or HFO/NIV were also more likely to receive any COVID-19 treatment and corticosteroids than Black patients. There were no further statistically significant differences in COVID-19 treatments in Black compared with Asian patients. aORs comparing treatment use in Black patients compared to patients with a recorded race of Other were highly heterogenous but generally indicated that patients with a recorded race of Other were more likely to receive COVID-19 treatments than Black patients.

Table 2 presents baseline characteristics overall and by ethnicity. Hispanic patients were, younger (median age: 57 vs. 65 years), less likely to be White (65% vs. 71%) and had a lower comorbidity burden than non-Hispanic patients (CCI ≥ 4: 14% vs. 20%). The proportion of patients receiving any COVID-19 treatment within 2 days of admission was similar across each ethnic grouping (Hispanic: 86%, non-Hispanic: 86%, Unknown: 85%). Hispanic patients were slightly more likely to receive corticosteroids than non-Hispanic patients and patients with unknown ethnicity (Hispanic: 54%, non-Hispanic: 52%, Unknown: 47%).

**Table 2:** Baseline characteristics of study cohort overall and according to ethnicity

		Overall	Hispanic	Non-Hispanic	Unknown
# Patients		**454,761**	**74,199**	**329,940**	**50,622**
Age (years)	Median (IQR)	64 (52–75)	57 (45–70)	65 (54–76)	65 (53–76)
Age group	18–49 yrs	95,726 (21%)	24,832 (34%)	60,683 (18%)	10,211 (20%)
	50–64 yrs	137,891 (30%)	24,253 (33%)	98,731 (30%)	14,907 (29%)
	65 + yrs	221,144 (49%)	25,114 (34%)	170,526 (52%)	25,504 (50%)
Gender	Male	235,938 (52%)	40,240 (54%)	169,028 (52%)	26,670 (53%)
Race	White	317,928 (70%)	48,392 (65%)	235,449 (71%)	34,087 (67%)
	Black	76,715 (17%)	1209 (2%)	69,085 (21%)	6421 (13%)
	Asian	9297 (2%)	193 (0%)	8017 (2%)	1087 (2%)
	Other^1^	50,821 (11%)	24,405 (33%)	17,389 (5%)	9027 (18%)
Primary payer	Commercial	130,807 (29%)	22,518 (30%)	95,035 (29%)	13,254 (26%)
	Medicare	229,982 (51%)	25,038 (34%)	178,729 (54%)	26,215 (52%)
	Medicaid	49,919 (11%)	12,187 (16%)	30,661 (9%)	7071 (14%)
	Other payer	44,053 (10%)	14,456 (20%)	25,515 (8%)	4082 (8%)
Hospital location	Urban	390,518 (86%)	69,281 (93%)	279,738 (85%)	41,499 (82%)
	Rural	64,243 (14%)	4918 (7%)	50,202 (15%)	9123 (18%)
Hospital bed size	< 100	32,519 (7%)	2806 (4%)	26,536 (8%)	3177 (6%)
	100–199	77,214 (17%)	16,312 (22%)	54,557 (17%)	6345 (13%)
	200–299	91,302 (20%)	11,431 (15%)	66,999 (20%)	12,872 (25%)
	300–399	87,621 (19%)	10,307 (14%)	66,397 (20%)	10,917 (22%)
	400–499	43,399 (10%)	7830 (11%)	31,089 (9%)	4480 (9%)
	> = 500	122,706 (27%)	25,513 (34%)	84,362 (26%)	12,831 (25%)
Hospital teaching status	Yes	178,769 (39%)	32,667 (44%)	124,831 (38%)	21,271 (42%)
Hospital region	Midwest	96,899 (21%)	6121 (8%)	81,817 (25%)	8961 (18%)
	Northeast	43,196 (10%)	5752 (8%)	29,448 (9%)	7996 (16%)
	South	251,394 (55%)	49,517 (67%)	185,226 (56%)	16,651 (33%)
	West	63,272 (14%)	12,809 (17%)	33,449 (10%)	17,014 (34%)
Admission month	Pre-Delta	273,143 (60%)	48,156 (70%)	188,110 (57%)	36,877 (73%)
	Delta	150,997 (33%)	22,680 (31%)	117,080 (36%)	11,237 (22%)
	Omicron	30,621 (7%)	3363 (5%)	24,750 (8%)	2508 (5%)
CCI	0	138,559 (31%)	27,880 (38%)	95,593 (29%)	15,086 (30%)
	1 to 3	228,941 (50%)	35,923 (48%)	166,964 (51%)	26,054 (56%)
	≥ 4	87,261 (19%)	10,396 (14%)	67,383 (20%)	9482 (19%)
Key comorbidities	Immunocompromised condition	47,643 (11%)	6061 (8%)	36,582 (11%)	5000 (9%)
	Renal disease	87,370 (19%)	9906 (13%)	67,831 (21%)	9633 (19%)
Baseline supplemental oxygen requirements	NSOc	201,934 (44%)	34,825 (47%)	139,956 (42%)	27,153 (54%)
	LFO	173,143 (38%)	27,859 (38%)	130,047 (39%)	15,237 (30%)
	HFO/NIV	67,086 (15%)	9396 (13%)	50,893 (15%)	6797 (13%)
	IMV	12,598 (3%)	2119 (3%)	9044 (3%)	1435 (3%)
Baseline ICU use	Yes	82,793 (18%)	18,872 (25%)	57,634 (18%)	6287 (12%)
COVID-19 treatment initiation upon hospital admission	Any COVID-19 treatment	390,344 (86%)	63,792 (86%)	283,779 (86%)	42,773 (85%)
	Corticosteroids	380,622 (84%)	62,209 (84%)	276,501 (84%)	41,912 (83%)
	Remdesivir	234,386 (52%)	39,860 (54%)	170,565 (52%)	23,961 (47%)
	Baricitinib	16,378 (4%)	1964 (3%)	13,345 (4%)	1069 (2%)
	Tocilizumab	17,928 (4%)	3282 (4%)	13,365 (4%)	1281 (3%)

^1^Other race includes race designations that have been rolled into “other” to ensure that the dataset confirms to regulatory requirements as well as race designations of “unable to determine.” See Supplementary Table 1 for variable definitions (i.e., renal disease, immunocompromised condition)

*CCI*, Charlson Comorbidity Index; *IQR*, inter-quartile range; *NSOc*, supplemental oxygen charges; *LFO*, low-flow oxygen; *HFO/NIV*, high-flow oxygen/non-invasive ventilation; *IMV*, invasive mechanical ventilation

The proportion of patients receiving any COVID-19 treatments by ethnicity and baseline supplemental oxygen requirement is presented in Supplementary Table 2. The proportion of NSOc patients receiving any COVID-19 treatments was similar regardless of patient ethnicity (Hispanic: 77.3%, non-Hispanic: 75.5%, Unknown: 77.2%). This finding was also noted in patients requiring LFO (Hispanic: 93.2%, non-Hispanic: 93.2%, Unknown: 91.7%), HFO/NIV (Hispanic: 96.1%, non-Hispanic: 95.6%, Unknown: 96.3%), and IMV (Hispanic: 89.1%, non-Hispanic: 90.9%, Unknown: 89.8%) at baseline. Hispanic patients on NSOc or HFO/NIV were more likely to initiate remdesivir than non-Hispanic patients.

**Fig. 3:**
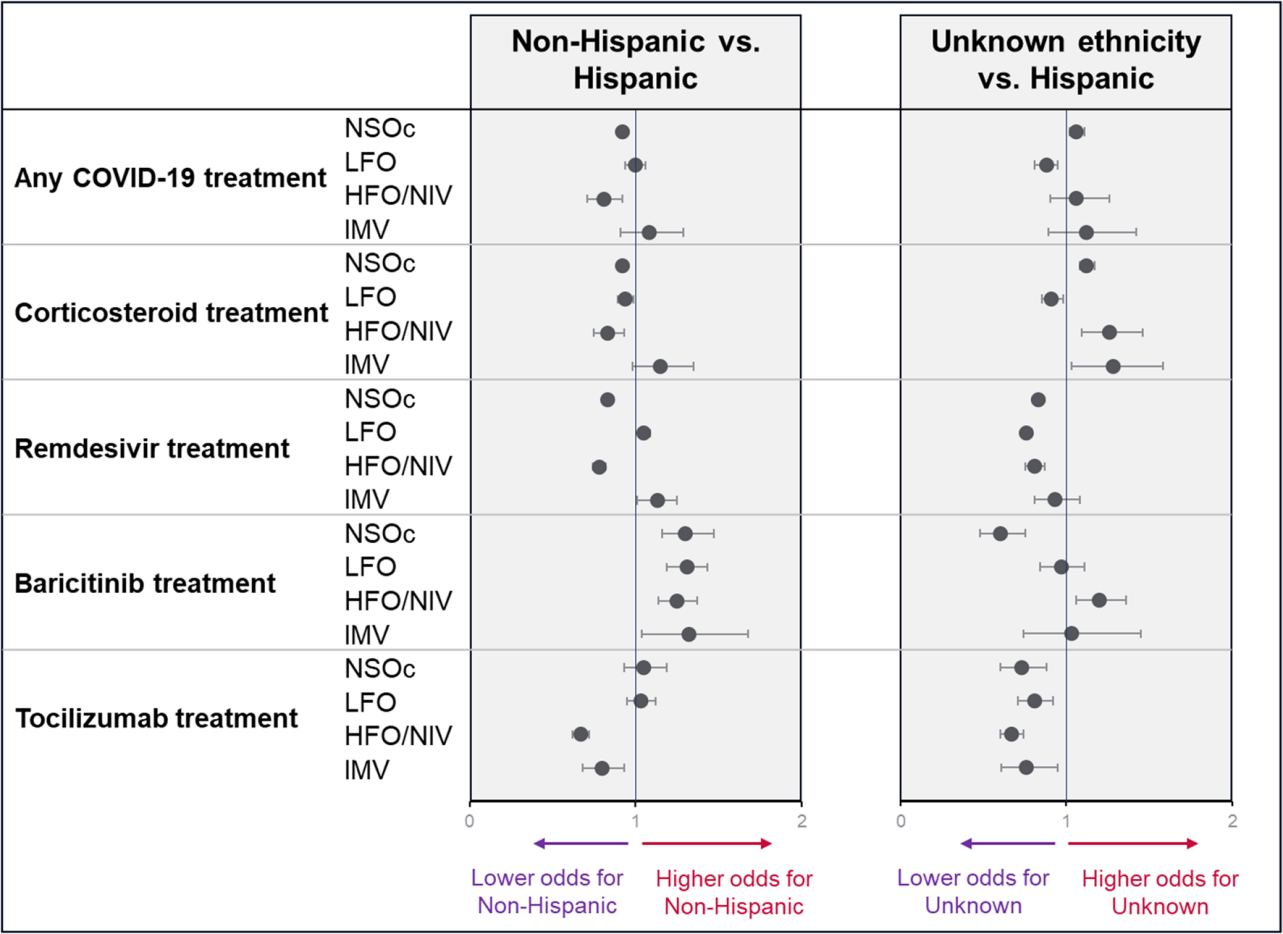
Likelihood of receiving COVID-19 treatment upon hospital admission by ethnicity (adjusted multivariable regression model). Model adjusted for age group, gender, race, hospital bed size, hospital location, teaching hospital, hospital region, payer type, variant period, CCI categories, ICU use at baseline, renal disease at baseline, immunocompromised condition at baseline. *NSOc: no supplemental oxygen charges; LFO: low-flow oxygen; HFO/NIV: high-flow oxygen/non-invasive ventilation; IMV: invasive mechanical ventilation*

Differences in the administration of COVID-19 treatment between Hispanic and non-Hispanic patients varied considerably according to treatment type and baseline supplemental oxygen requirement after multivariable adjustment (Fig. 3, Supplementary Table 2). Patients with unknown ethnicity were significantly more likely to receive tocilizumab than Hispanic patients across all levels of supplemental oxygen requirement (NSOc aOR: 0.73 (0.60–0.88), LFO: 0.81 (0.71–0.92), HFO/NIV: 0.67 (0.60–0.74), IMV: 0.76 (0.61–0.95)).

## Discussion

### Key Findings

In this study including 454,761 hospitalized COVID-19 patients from across the US, Black patients were less likely to receive COVID-19 treatment compared to White and Asian patients, even after accounting for differences in comorbidity burden and demographics. This finding was consistent across all levels of COVID-19 severity, as measured using baseline supplemental oxygen requirement. These findings are indicative of both under- and overtreatment according to patient race. For example, White patients on NSOc were significantly more likely to receive corticosteroids and baricitinib than Black patients, despite clinical guidelines recommending against initiating these treatments in patients on NSOc. Conversely, White patients requiring LFO were more likely to receive remdesivir treatment than Black patients, which was indicative of undertreatment of Black patients. In fact, White patients had higher odds of receiving remdesivir than Black patients across the COVID-19 spectrum in hospitalized patients. While remdesivir is selectively recommended for initiation among patients requiring HFO/NIV, growing evidence indicates its likely beneficial role among patient patients with and without hypoxemia [13, 16]. The finding of inequitable delivery of life-saving treatments is likely to be an important contributor to the well-documented racial disparities in COVID-19 outcomes.

Few differences in the administration of COVID-19 treatments were observed between Hispanic and non-Hispanic patient populations, with the exception of tocilizumab. This lack of consistent trend in treatment initiation according to ethnicity is likely to reflect the considerable heterogeneity of the non-Hispanic and Hispanic patient populations.

We postulate that Tocilizumab was more accessible and readily used for all groups including black patients for a few reasons. Firstly, the one-time dosing meant providers may start it knowing they did not need to commit to a 5 or 10 days of treatment duration as they would RDV or Dexamethasone respectively. Additionally, the fact that it was not renally cleared means that even in patients with renal impairment (more commonly seen in Black patients), this was still viable treatment option.

### Relation to Other Studies

Stark racial disparities in morbidity and mortality have been documented for almost all health outcomes in the US, including for COVID-19 [17–20]. The findings from this study largely align with findings from a large study conducted using data from the Veterans Health Administration health care system [21]. Compared to White patients, Black patients were less likely to receive steroids (within-center aOR: 0.88, 95% CI: 0.80–0.96; between-center aOR: 0.67, 0.48–0.96), remdesivir (within-center aOR: 0.89, 0.83–0.95; between-center aOR: 0.68, 0.47–0.99), or immunomodulatory drugs (within-center aOR: 0.77, 0.67–0.87). The authors conclude that in addition to differences in health care access and exposure risk, differences in quality of COVID-19–specific treatments may contribute to adverse outcomes among minoritized patients. Our findings build upon these data by presenting data from a different, more generalizable patient population and stratified according to baseline oxygen supplemental oxygen requirement.

In a study using PCORnet data from the US National Patient-Centered Clinical Research Network, White inpatients were more likely to receive dexamethasone than Black, Asian, and other race inpatients (35.8% vs. 33.8% vs. 31.4% vs. 34.2%), though these differences were not statistically significant. In contrast, Black inpatients were more likely to receive remdesivir than White inpatients. However, these estimates were not adjusted for patient baseline characteristics, a likely explanation for the lack of alignment with the present study [22]. In another study in which estimates were adjusted for age, sex, and comorbidity status, Black inpatients were less likely to receive remdesivir than their White counterparts (aOR: 0.88, 95% confidence interval: 0.80, 0.96), though this disparity lessened as the pandemic progressed [23].

There are numerous potential explanations for the observed race disparity. First, barriers to healthcare, such as reduced access to testing and health insurance coverage may have contributed to delayed presentation to hospitals by Black patients [24]. However, there were no clear differences in baseline supplemental oxygen requirement and therefore disease severity at admission by race or ethnicity. Furthermore, findings were stratified according to baseline supplemental oxygen requirement, indicating that delayed presentation is unlikely to explain the observed racial disparities in administered treatments. Second, the observed race disparity may, in part, relate to differences in comorbidity burden according to patient race due to the downstream, wide-reaching impacts of structural racism. For example, equitable access to healthy, nutritious foods is essential for preventing chronic illnesses like cardiovascular disease, diabetes, and kidney disease. However, systemic barriers contribute to disparate rates of food insecurity along racial, educational, and economic lines [25]. In the present study, Black patients were more likely to have renal disease and immunocompromised conditions than White patients. This may have impacted the treatments administered to these patients due to concerns surrounding use of drugs such as remdesivir among patients with renal disease and the intensifying background immunosuppression with corticosteroids among immunocompromised patients [26]. Though renal disease and immunocompromised conditions were adjusted for in the analyses, residual confounding by comorbidity burden may have contributed to the observed disparities.

Lastly, another potential explanation for the inequitable delivery of COVID-19 treatments includes the possible persistent overestimation of arterial oxygen saturation among Asian, Black, and Hispanic individuals using pulse oximetry, as observed in a recent US-based study [27]. In this previous study, the overestimation of patient oxygenation status led to systematic failures in the identification of Black and Hispanic patients who were qualified for COVID-19 therapies and subsequent delays in COVID-19 therapy initiation. However, this inaccuracy in the evaluation of patient oxygenation status would not explain the observed overtreatment with corticosteroids of White patients on NSOc. An alternative explanation for the observed disparities is the potential for healthcare provider implicit bias, whereby there is a dissociation between provider attitudes and beliefs and the unconscious influence of negative implicit associations, leading to inequitable healthcare delivery [28]. Recognizing and raising awareness of the potential for implicit biases and its likely role in widening health inequities is an essential step to help to mitigation. Managing and reducing the impact of implicit bias will require targeted strategies including enhanced medical education and improving provider adherence to medical guidelines.

### Strengths

The study included a large population of hospitalized patients and was limited to patients with a primary diagnosis code for COVID-19. As a result, the study population primarily included patients admitted *for* COVID-19 rather than merely hospitalized incidentally *with* COVID-19 but hospitalized for a different primary reason. The study accounted for differences in COVID-19 severity according to race and ethnicity by stratifying findings according to baseline supplemental oxygen requirement. This was particularly important for two reasons. First, treatment recommendations for hospitalized COVID-19 patients are dependent on baseline supplemental oxygen requirement, yet the majority of earlier studies examining racial and ethnic disparities in COVID-19 treatments do not present findings according to baseline supplemental oxygen requirement. Second, evidence indicates that ethnic minority patients are more likely to have delayed presentation to healthcare providers due to being underinsured and prior negative experiences with the healthcare system, leading to mistrust and net lower levels of health insurance [29, 30]. Delays in presentation could contribute to heightened severity of COVID-19 at hospitalization among ethnic minority groups. Stratifying findings by baseline oxygen requirement was therefore important in case of heterogeneity of COVID-19 severity at presentation according to race or ethnicity, and wholly aligns with guideline recommendations for tiered therapy. Lastly, to account for the wide-ranging temporal variations in patient case-mix and patient management practices during the study period, the analyses were adjusted for the variant time period of hospital admission.

### Weaknesses

As with all real-world data research, there is the potential for residual confounding by, for example, socioeconomic status. While the CARES Act coverage was intended to help minimize disparities, the effect was incomplete, coverage was transient, and patients likely had lower awareness of this temporary safety net relative to providers’ awareness. By accounting for a large number of factors that cluster within socioeconomic status groups, including comorbidities and primary payer, we have attempted to minimize the risk of residual confounding by socioeconomic status. Other social factors such as a crowded home environment, the greater likelihood of COVID exposure as essential workers (grocery, home health aides, the inability to take sick time from work, etc.) may also have played a role but could not be assessed in this database. The database also does not capture information on patient vaccination status, treatments administered in outpatient settings, or time since symptom onset. While patient vaccination status is unlikely to have impacted treatments administered during patient hospitalization, treatments administered in outpatient settings and time since symptom onset may have influenced prescribing behaviors. Furthermore, there is some evidence demonstrating racial disparities in the administration of COVID-19 treatments in outpatient settings. This limitation should be considered when interpreting the results of this study [8, 22]. Lastly, given the acuity of patients’ illness and nature of the dataset, there is unavoidable heterogeneity of demographics being self-identified and externally-applied. The unknown race and other ethnic groups, in particular, are likely to be highly heterogeneous making the interpretation of findings relating to these group challenging.

### Implications

As we enter the endemic phase, it is crucial that we highlight persistent disparities in patient management and strive toward standardized care for all patients during hospitalization for COVID-19, regardless of racial and ethnic background. Raising awareness of these disparities to policymakers, clinicians, and clinical guideline developers through a call to action is a key step in preventing treatment disparities and improving patient outcomes. Demonstrating this issue to policymakers is particularly important to guide the development of strategies and programs aimed at achieving more equitable COVID-19 treatment. Eliminating racial disparities in health is an urgent public health priority and eliminating structural racism itself is necessary to fully achieve health equity. For example, tackling food insecurity, a social driver of health, represents an opportunity to improve health by addressing root causes instead of downstream symptoms. Creating conditions for all communities to access affordable produce, reducing reliance on highly processed items, and dismantling policies that propagate food apartheid could reduce preventable nutrition-related chronic diseases. Working to close these equity gaps through both system-level reforms and frontline practices is necessary for evidence-based, equitable medicine even beyond the COVID-19 pandemic.

We recognize that relying on any single strategy or program will be insufficient as dismantling systemic and institutional racism will require comprehensive reforms across multiple sectors of society. Nevertheless, small steps such as protocoled computer-based order entry sets (“order bundles”) objectively triggered by flowsheet data within the electronic medical record is one means by which acts of omission can be minimized for all. Controlling overuse is more challenging, as other reasons for use of immunomodulatory agents and “alert fatigue” would pose operational challenges.

Ensuring equitable access to evidence-based therapies benefits all of US society by ensuring patients who are likely to benefit receive appropriate, potentially lifesaving therapies.

## Supplementary Information

Below is the link to the electronic supplementary material.

Supplementary file1 (DOCX 60 KB)

## Data Availability

The data that support the findings of this study are available from Premier, Inc. (https://www.premierinc.com/). Restrictions apply to the availability of these data, which were used under license for this study.
