# Supplementary material for: Racial and Ethnic Disparities in COVID-19 Treatments in the United States
Source: J Racial Ethn Health Disparities. 2024 Feb 26;12(2):1052–62. doi: 10.1007/s40615-024-01942-0 (PMC11914345; doi:10.1007/s40615-024-01942-0)
Supplement: Supplementary file 1 — Supplementary file1 (DOCX 60 KB) [file 40615_2024_1942_MOESM1_ESM.docx]

# Supplementary Materials

**Supplementary Table 1.** Study Variable Definitions

| Key Study Variables | | Definitions |
| --- | --- | --- |
| Comorbidities | Renal disease | ICD-10-CM diagnosis codes: I12.0, I13.1, N03.2, N03.3, N03.4, N03.5, N03.6, N03.7, N05.2, N05.3, N05.4, N05.5, N05.6, N05.7, N18.x, N19, N25.0, Z49.0, Z49.1, Z49.2, Z94.0, Z99.2 |
|  | Immunocompromised conditions | Yes / No, defined as a diagnosis record of an ICD-10-CM code for transplant (Z94.x) or hematologic malignancies (C81.x, C82.x, C83.x, C84.x, C85.x, C88.x, C90.x, C91.x, C92.x, C93.x, C94.x, C95.x, C96.x) or primary immunodeficiencies (D80.x, D81.x, D82.x, D83.x, D84.x, G11.3, E70.330, D71.x, D70.x), asplenia (Q89.01, Z90.81), chronic kidney disease (N18.x), chronic liver disease or cirrhosis (K76.9, K74.60), toxic effects of antineoplastics (T45.1x), bone marrow failure/aplastic anemia (D61.x), severe combined immunodeficiencies (D80.x, D81.x, D82.x, D83.x, D84.x, D86.x, D89.0, D89.1, D89.2, D89.3 , D89.4x, D89.81, D89.82, D89.89, D89.9), HIV (B20), Post-hematopoietic cell transplant recipients who have chronic graft-versus-host disease or who are taking immunosuppressive medications for another indication (Z89.8x, Z79.52, Z79.61, Z79.62x, Z79.63x, Z79.64, Z79.69, Z79.810, Z79.811, Z79.818) |
|  | Charlson Comorbidity Index | See *Glasheen, W.P., et al., Charlson Comorbidity Index: ICD-9 Update and ICD-10 Translation. Am Health Drug Benefits, 2019. 12(4): p. 188-197* |
| Supplemental oxygen  requirements | IMV | Billing charges: invasive mechanical ventilation, tracheostomy, endotracheal tube, intubation |
|  | HFO/NIV | Billing charges: negative-pressure ventilation, positive-pressure ventilation, CPAP, BiPAP, high flow system via nasal cannula, venturi face mask, rebreather, non-rebreather mask, positive expiratory pressure |
|  | LFO | Billing charges at baseline: Simple face mask, oxygen pendant, low-flow system via nasal cannula, oxygen supply |
|  | NSOc | No billing charges for IMV or ECMO, HFO/NIV, or LFO at baseline (defined above) |
| COVID-19 treatments | Anticoagulants | Billing for treatment: Treatments used at baseline: apixaban, argatroban, desirudin, lepirudin, dabigatran, danaparoid, edoxaban, tinzaparin, heparin (excluding use of heparin flush), ardeparin, bivalirudin |
|  | Baricitinib | Billing for treatment: Baricitinib; ICD-10 procedure codes (effective Jan 1 2021): XW0DXM6, XW0H7M6, XW0G7M6 |
|  | Corticosteroids | Billing for treatment: prednisone, prednisolone, methylprednisolone, hydrocortisone, dexamethasone |
|  | Convalescent plasma | Billing for treatment: convalescent plasma; ICD-10 procedure codes (effective Aug 1 2020): XW13325, XW14325 |
|  | Tocilizumab | Billing for treatment: Tocilizumab; ICD-10 procedure codes (effective Aug 1 2020): XW033H5, XW043H5 |

**Supplementary Table 2.** Proportion of patients initiating COVID-19 treatments according to ethnicity, race and baseline supplemental oxygen requirement

|  | **Overall** | **Race** | | | | **Ethnicity** | | |
| --- | --- | --- | --- | --- | --- | --- | --- | --- |
|  |  | **White** | **Black** | **Asian** | **Other^1^** | **Hispanic** | **Non-Hispanic** | **Unknown** |
|  | **454,761** | **317,928** | **76,715** | **9,297** | **50,821** | **74,199** | **329,940** | **50,622** |
| **NSOc at baseline** | **n=201934** | **n=135059** | **n=40276** | **n=4402** | **n=22197** | **n=34825** | **n=139956** | **n=27153** |
| Any COVID-19 treatment | 76.0% | 77.9% | 71.2% | 74.9% | 73.4% | 77.3% | 75.5% | 77.2% |
| Corticosteroids | 73.3% | 75.4% | 68.6% | 71.1% | 69.9% | 74.5% | 72.7% | 75.1% |
| Remdesivir | 41.8% | 43.9% | 34.8% | 45.0% | 40.7% | 45.9% | 40.9% | 41.1% |
| Baricitinib | 1.3% | 1.4% | 0.9% | 0.5% | 1.0% | 1.2% | 1.4% | 0.4% |
| Tocilizumab | 1.1% | 1.1% | 1.0% | 1.0% | 1.1% | 1.3% | 1.1% | 0.6% |
|  |  |  |  |  |  |  |  |  |
| **LFO at baseline** | **n=173143** | **n=126067** | **n=23789** | **n=3366** | **n=19921** | **n=27859** | **n=130047** | **n=15237** |
| Any COVID-19 treatment | 93.1% | 93.7% | 90.5% | 92.8% | 92.5% | 93.2% | 93.2% | 91.7% |
| Corticosteroids | 91.6% | 92.2% | 89.1% | 90.9% | 90.7% | 92.0% | 91.6% | 90.5% |
| Remdesivir | 57.5% | 58.6% | 50.7% | 62.4% | 58.2% | 58.2% | 58.2% | 50.6% |
| Baricitinib | 3.6% | 3.8% | 3.1% | 1.8% | 2.9% | 2.5% | 4.0% | 2.1% |
| Tocilizumab | 3.6% | 3.5% | 4.3% | 3.8% | 3.5% | 3.7% | 3.7% | 2.3% |
|  |  |  |  |  |  |  |  |  |
| **HFO/NIV at baseline** | **n=67086** | **n=48543** | **n=10398** | **n=1224** | **n=6921** | **n=9396** | **n=50893** | **n=6797** |
| Any COVID-19 treatment | 95.8% | 96.3% | 93.6% | 98.2% | 95.1% | 96.1% | 95.6% | 96.3% |
| Corticosteroids | 93.8% | 94.3% | 92.0% | 96.1% | 92.6% | 93.9% | 93.6% | 95.1% |
| Remdesivir | 65.7% | 66.4% | 60.0% | 74.6% | 67.7% | 71.1% | 64.7% | 65.2% |
| Baricitinib | 10.0% | 10.8% | 7.4% | 8.0% | 8.9% | 8.1% | 10.6% | 8.4% |
| Tocilizumab | 11.6% | 11.4% | 11.6% | 12.7% | 12.7% | 15.8% | 11.1% | 9.1% |
|  |  |  |  |  |  |  |  |  |
| **IMV at baseline** | **n=12598** | **n=8259** | **n=2252** | **n=305** | **n=1782** | **n=2119** | **n=9044** | **n=1435** |
| Any COVID-19 treatment | 90.5% | 91.2% | 88.4% | 89.5% | 90.0% | 89.1% | 90.9% | 89.8% |
| Corticosteroids | 87.7% | 88.4% | 86.0% | 87.5% | 86.3% | 85.2% | 88.3% | 87.5% |
| Remdesivir | 50.2% | 51.7% | 45.3% | 56.7% | 48.4% | 47.4% | 51.6% | 46.1% |
| Baricitinib | 7.5% | 8.5% | 5.5% | 5.2% | 6.2% | 5.3% | 8.5% | 4.7% |
| Tocilizumab | 13.9% | 13.8% | 14.5% | 12.1% | 13.8% | 14.8% | 14.2% | 10.6% |

^1^Other race includes race designations that have been rolled into “other” to ensure that the dataset confirms to regulatory requirements as well as race designations of “unable to determine”

**Supplementary Table 3.** Adjusted odds ratios for initiation of COVID-19 treatments within two days according to race and ethnicity, stratified by baseline supplemental oxygen requirement

| **Treatment** | **Baseline supplemental oxygen requirements** | **OR [95% CI] for initiation of treatment within two days of hospitalization** | | | | |
| --- | --- | --- | --- | --- | --- | --- |
|  |  | **Race** | | | **Ethnicity** | |
|  |  | **White vs. Black** | **Asian vs. Black** | **Other vs. Black** | **Non-Hispanic vs. Hispanic** | **Unknown vs. Hispanic** |
| Any COVID-19 treatment | NSOc | 1.33 [1.29 - 1.36] | 1.19 [1.10 - 1.28] | 1.04 [1.00 - 1.08] | 0.92 [0.89 - 0.95] | 1.06 [1.02 - 1.11] |
|  | LFO | 1.47 [1.40 - 1.55] | 1.35 [1.17 - 1.56] | 1.21 [1.12 - 1.30] | 1.00 [0.94 - 1.06] | 0.88 [0.81 - 0.95] |
|  | HFO/NIV | 1.43 [1.30 - 1.58] | 3.39 [2.19 - 5.24] | 1.00 [0.86 - 1.17] | 0.81 [0.71 - 0.92] | 1.06 [0.90 - 1.26] |
|  | IMV | 1.31 [1.11 - 1.54] | 1.31 [0.87 - 1.96] | 1.34 [1.06 - 1.68] | 1.08 [0.91 - 1.29] | 1.12 [0.89 - 1.42] |
| Corticosteroid | NSOc | 1.33 [1.30 - 1.37] | 1.14 [1.06 - 1.23] | 1.02 [0.98 - 1.06] | 0.92 [0.89 - 0.95] | 1.12 [1.08 - 1.17] |
|  | LFO | 1.43 [1.36 - 1.50] | 1.23 [1.08 - 1.40] | 1.10 [1.02 - 1.18] | 0.94 [0.89 - 0.99] | 0.91 [0.85 - 0.98] |
|  | HFO/NIV | 1.26 [1.15 - 1.37] | 2.12 [1.57 - 2.88] | 0.89 [0.78 - 1.01] | 0.83 [0.75 - 0.93] | 1.26 [1.09 - 1.46] |
|  | IMV | 1.25 [1.08 - 1.45] | 1.37 [0.94 - 1.99] | 1.20 [0.97 - 1.47] | 1.15 [0.98 - 1.35] | 1.28 [1.03 - 1.58] |
| Remdesivir | NSOc | 1.33 [1.29 - 1.36] | 1.39 [1.30 - 1.48] | 1.06 [1.03 - 1.11] | 0.83 [0.81 - 0.85] | 0.83 [0.80 - 0.86] |
|  | LFO | 1.32 [1.28 - 1.36] | 1.54 [1.43 - 1.67] | 1.30 [1.25 - 1.36] | 1.05 [1.02 - 1.09] | 0.76 [0.73 - 0.79] |
|  | HFO/NIV | 1.20 [1.15 - 1.26] | 1.79 [1.56 - 2.06] | 1.09 [1.01 - 1.17] | 0.78 [0.74 - 0.82] | 0.81 [0.75 - 0.87] |
|  | IMV | 1.25 [1.13 - 1.38] | 1.71 [1.33 - 2.20] | 1.19 [1.04 - 1.37] | 1.13 [1.01 - 1.25] | 0.93 [0.81 - 1.08] |
| Baricitinib | NSOc | 1.54 [1.37 - 1.73] | 0.82 [0.53 - 1.29] | 1.64 [1.37 - 1.97] | 1.30 [1.16 - 1.47] | 0.60 [0.48 - 0.75] |
|  | LFO | 1.15 [1.05 - 1.25] | 0.76 [0.57 - 1.00] | 1.15 [1.02 - 1.31] | 1.31 [1.19 - 1.43] | 0.97 [0.84 - 1.11] |
|  | HFO/NIV | 1.33 [1.21 - 1.45] | 1.05 [0.82 - 1.33] | 1.22 [1.07 - 1.38] | 1.25 [1.14 - 1.37] | 1.20 [1.06 - 1.36] |
|  | IMV | 1.36 [1.09 - 1.69] | 0.90 [0.50 - 1.61] | 1.28 [0.94 - 1.74] | 1.32 [1.04 - 1.68] | 1.03 [0.74 - 1.45] |
| Tocilizumab | NSOc | 1.16 [1.03 - 1.31] | 1.15 [0.83 - 1.59] | 1.21 [1.02 - 1.44] | 1.05 [0.93 - 1.19] | 0.73 [0.60 - 0.88] |
|  | LFO | 0.81 [0.75 - 0.87] | 1.15 [0.94 - 1.39] | 0.91 [0.81 - 1.01] | 1.03 [0.95 - 1.12] | 0.81 [0.71 - 0.92] |
|  | HFO/NIV | 0.90 [0.83 - 0.96] | 1.26 [1.04 - 1.52] | 0.90 [0.81 - 1.00] | 0.67 [0.62 - 0.72] | 0.67 [0.60 - 0.74] |
|  | IMV | 0.87 [0.75 - 1.01] | 0.97 [0.66 - 1.43] | 0.95 [0.77 - 1.16] | 0.80 [0.68 - 0.93] | 0.76 [0.61 - 0.95] |
